# The neurodynamical basis of multi-item working memory capacity: sequential vs simultaneous stimulation paradigms

**DOI:** 10.1186/1471-2202-16-S1-P58

**Published:** 2015-12-18

**Authors:** Marta Balagué, Laura Dempere-Marco

**Affiliations:** 1 Moisès Broggi Hospital, Consorci Sanitari Integral, Sant Joan Despí, 08970, Spain; 2 Department of Information and Communication Technologies, Universitat Pompeu Fabra, Barcelona, 08018, Spain

## 

When investigating multi-item WM, and in contrast to single item experiments, a decision must be made regarding a key aspect of the stimulation protocol: how the memory set is presented to the subject *simultaneously *or *sequentially*. It is worth noting that most studies investigating multi-item WM do not address this issue and focus either in simultaneous stimulation protocols (e.g. [1,2]) or in sequential stimulation protocols (e.g. [3]) without confronting the two situations. This is nevertheless an aspect which provides a benchmark to probe and compare the different theories regarding how resources are allocated among the different items of a memory set [4,5]. In this study, we explore a biophysically-realistic attractor model of visual working memory (VWM) endowed with synaptic facilitation and investigate what are the effects of varying the dynamics of the facilitation process. We find that: 1) it is possible to reproduce experimentally observed effects such as the *recency *effect in sequential stimulation protocols (i.e. items presented in the final positions of a sequence are more likely to be retained in WM), and 2) WM capacity is boosted in both sequential and stimulation protocols when endowing the attractor network with synaptic facilitation.

## Conclusions

In agreement with our previous results [2], synaptic facilitation boosts the WM capacity limit by effectively increasing the synaptic strengths just for those pools to which a cue is applied, and then maintaining the synaptic facilitation by the continuing neuronal firing in only these pools when the cue is removed. In this study, the time constant τ_F _of the synaptic facilitation process has been found to play an important role in modulating this effect with large τ_F _values leading to larger capacity limits in both sequential and simultaneous stimulation protocols. However, too large τ_F _values lead to neuronal dynamics which are not compatible with the *recency *effect, thus constraining the range of values that τ_F _may take.

**Figure 1:**
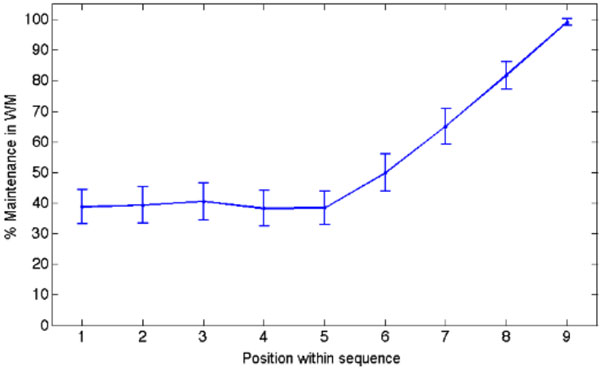
**Maintenance of an item in WM memory as a function of its position within a sequence**. The results are derived from computational simulations (100 blocks of 100 trials) of a delayed match-to-sample task (same stimulation protocol as in [3] and test item assimilated to a delayed match-to-sample task) with 9 selective neural assemblies sequentially stimulated. Maintenance in WM is estimated by assuming that an item is held in memory when its associated selective pool shows a mean persistent activity ν ≥ 30 Hz during a period of 500 ms 2 s after the end of the last stimulation. The network parameters can be found in [2] and τ_F_=750 ms in this example.
